# Transcriptome reprogramming of resistant and susceptible peach genotypes during *Xanthomonas arboricola* pv. *pruni* early leaf infection

**DOI:** 10.1371/journal.pone.0196590

**Published:** 2018-04-26

**Authors:** Fabio Gervasi, Patrizia Ferrante, Maria Teresa Dettori, Marco Scortichini, Ignazio Verde

**Affiliations:** Consiglio per la ricerca in agricoltura e l’analisi dell’economia agraria (CREA) – Centro di Olivicoltura, Frutticoltura e Agrumicoltura (CREA-OFA), Rome, Italy; Estacion Experimental del Zaidin—CSIC, SPAIN

## Abstract

Bacterial spot caused by *Xanthomonas arboricola* pv. *pruni* (Xap) is a major threat to *Prunus* species worldwide. The molecular mechanisms of peach resistance to Xap during early leaf infection were investigated by RNA-Seq analysis of two *Prunus persica* cultivars, ‘Redkist’ (resistant), and ‘JH Hale’ (susceptible) at 30 minutes, 1 and 3 hours-post-infection (hpi). Both cultivars exhibited extensive modulation of gene expression at 30 mpi, which reduced significantly at 1 hpi, increasing again at 3 hpi. Overall, 714 differentially expressed genes (DEGs) were detected in ‘Redkist’ (12% at 30 mpi and 1 hpi and 88% at 3 hpi). In ‘JH Hale’, 821 DEGs were identified (47% at 30 mpi and 1 hpi and 53% at 3 hpi). Highly up-regulated genes (fold change > 100) at 3 hpi exhibited higher fold change values in ‘Redkist’ than in ‘JH Hale’. RNA-Seq bioinformatics analyses were validated by RT-qPCR. In both cultivars, DEGs included genes with putative roles in perception, signal transduction, secondary metabolism, and transcription regulation, and there were defense responses in both cultivars, with enrichment for the gene ontology terms, ‘immune system process’, ‘defense response’, and ‘cell death’. There were particular differences between the cultivars in the intensity and kinetics of modulation of expression of genes with putative roles in transcriptional activity, secondary metabolism, photosynthesis, and receptor and signaling processes. Analysis of differential exon usage (DEU) revealed that both cultivars initiated remodeling their transcriptomes at 30 mpi; however, ‘Redkist’ exhibited alternative exon usage for a greater number of genes at every time point compared with ‘JH Hale’. Candidate resistance genes (*WRKY*-like, *CRK*-like, *Copper amine oxidase*-like, and *TIR-NBS-LRR*-like) are of interest for further functional characterization with the aim of elucidating their role in *Prunus* spp. resistance to Xap.

## Introduction

*Xanthomonas arboricola* pv. *pruni* (Xap) is the causal agent of bacterial spot on stone fruits and it is distributed widely across all continents. [1]. This bacterium affects all stone fruit crops, including *Prunus davidiana* and *Prunus mume* as well as the ornamental species *Prunus laurocerasus*, *Prunus japonica* and their derived hybrids [2,3]. Furthermore, Xap is included in the EPPO A2 quarantine list and European Council Directive 2000/29/EC [1,4] and it has potential to cause serious losses by reducing the marketability of fruit and weakening trees through its symptoms of leaf spots, defoliation, and lesions on twigs.

Peach [*Prunus persica* (L.) Batsch] is one of the most important fruit crops in temperate regions with a global annual production of 25 million tonnes (FAOSTAT 2016, http://www.fao.org/faostat/en/#data/QC). *P*. *persica* is a self-pollinating species with a small genome (approximatively 230 Mb) arranged on eight chromosomes. The *P*. *persica* genome sequence was obtained by an international consortium [5] and was recently improved [6], generating a high-quality genome sequence with a large portion of sequences mapped to chromosomes, high accuracy, and high contiguity, representing an important tool for functional and comparative genomics in the genus *Prunus*, as well as the *Rosaceae* family, due to its high quality [6,7].

Although field trials based on innovative bacterial spot control strategies, such as viral antagonism [8,9] or bio-control with the strain *Pseudomonas fluorescens* G19 [10], produced interesting results, breeding for resistance still remains the most effective strategy. A high degree of variability regarding the susceptibility of peach to Xap has been described, both in multi-year field observations and in laboratory tests [11–15], leading to a classification of peach cultivars as: susceptible (e.g., ‘O’Henry’, ‘Rich Lady’), moderately susceptible (e.g.,‘JH Hale’), moderately resistant (e.g., ‘Cresthaven’, ‘Harrow Diamond’, ‘Redhaven’), and highly resistant (e.g., ‘Candor’, ‘Clayton’, ‘Redkist’) [16]. Nevertheless, the genetic and molecular basis of resistance to Xap have only recently been investigated in the *Prunus* genus. In peach, a quantitative trait loci (QTL) mapping study detected 14 QTLs with additive effects on the resistance phenotype, four of which were major QTLs on linkage groups (LG) 1, 4, 5, and 6. Thirty nucleotide binding site-leucine rich repeat (*NBS-LRR*) genes were identified in these regions, highlighting the crucial role of R-genes in peach resistance [17]. In apricot (*Prunus armeniaca* L.), one major QTL was identified on the LG 5 and gene analysis in the QTL region identified six candidate genes encoding receptor-like kinase (RLK), LRR proteins, and disease resistance proteins putatively involved in plant resistance mechanisms [18]. Sherif and co-workers [19,20] reported modulation of the expression patterns of five pathogenesis-related (PR) genes and five ethylene response factors (ERFs) in the leaves of resistant (‘Venture’) and susceptible (‘BabyGold 5’) cultivars of peach after Xap artificial infection.

Plant-pathogen interactions involve rapid physiological responses immediately after infection. In *Arabidopsis thaliana* the first mRNA level changes have been described as early as 10 minutes after inoculation with *Pseudomonas syringae* pv. *tomato* [21], 30 minutes after elicitor contact [22,23], and 1 hour after bacterial infiltration [24]. In rice, transcriptomic remodeling was observed at 1 hour after infection with *Xanthomonas oryzae* pv. *oryzae* [25], and similar results were reported for tobacco challenged with *Xanthomonas axonopodis* pv. *citri* [26]. During the peach/Xap interaction, gene induction was observed at 1 hour-post-infection (hpi) [19,20]; furthermore, RNA-Seq analysis revealed that there were already significant changes in gene expression at 2 hpi after Xap artificial infection of the moderately susceptible peach rootstock, ‘GF305’ [27].

Transcriptome profiling is a valuable tool that provides a global view of gene expression in response to stimuli. This approach has revealed that plants challenged with pathogens use analogous sets of genes for both compatible and incompatible interactions, and that the kinetics and intensity of gene induction are crucial for plant outcomes [28–30]. Moreover, a number of RNA-Seq studies have provided information about global gene expression in host-pathogen interactions [31–35], as well as in host-*Xanthomonas* pathosystems [25,27,36–38]. Gene expression was also investigated at the transcriptome level to analyze the compatible interaction between peach plants with a moderately susceptible genotype and Xap at 2 and 12 hpi [27].

Although some major QTLs for resistance have been identified and despite the various molecular analyses performed to date, substantial research is still required to unravel the molecular mechanisms underlying the resistance and the susceptibility of *Prunus* species to Xap, particularly during the very early stages following infection.

The present article describes an RNA-Seq gene expression study which provides a global view of the transcriptome dynamics of a resistant (‘Redkist’) and a of a moderately susceptible (‘JH Hale’) cultivar of *P*. *persica*. Differentially expressed genes (DEGs) were determined at very early stages (30 mpi, 1 and 3 hpi) after Xap inoculation for each cultivar and the differences between the two defense responses are highlighted. In addition, to further evaluate transcriptome remodeling, differential exon usage (DEU) was also analyzed.

## Materials and methods

### Plant material, inoculation, and experimental design

Peach plants for the experimental use were obtained by grafting the cultivars ‘Redkist’ (resistant) and ‘JH Hale’ (moderately susceptible) onto the GF677 rootstock clone in winter. Cuttings were obtained from adult plants growing in the experimental orchards of the CREA-OFA (Centro Nazionale Germoplasma Frutticolo-CNGF), located in Rome (central Italy; latitude: from 41°47'49.79"N to 41°47'50.45"N; Longitude: from 12°33'50.69"E to 12°33'56.42"E). Sixteen grafted plants were trained in pots before inoculation and received standard fertilization and irrigation. After grafting (December) the trees were kept in 6 L pots for vegetative growth in a quarantine greenhouse until July, when they were moved to the infection chamber for a 3 day acclimatization period under controlled conditions (28°C; 80% RH; 16 h light); trees were kept under these conditions until sampling. ‘Redkist’ is a yellow melting flesh peach cultivar obtained from a mutation of ‘Redskin’ and is highly resistant to bacterial spot [16]. ‘JH Hale’ is a yellow melting flesh peach, obtained from self-pollination of ‘Elberta’ [39] and is moderately susceptible to Xap [16]. ‘JH Hale’ was chosen as the susceptible cultivar as, being one of the parental strains of the resistant cultivar [16], it was expected to exhibit a low background of transcriptomic differences, facilitating identification of changes specific to the response to infection.

The source of inoculum for the artificial infections was Xap NCPPB 2588, which was isolated from *P*. *persica* in South Africa in 1974, and it is virulent to peach after artificial inoculation [40]. For inoculum preparation, bacteria were cultured on glucose-yeast extract-calcium carbonate agar plates at 26°C for 48h and suspended in sterile saline (0.85% of NaCl in distilled water) to a concentration of 1–2 × 10^8^ colony-forming units (c.f.u.) ml^-1^. Eight trees of each cultivar (two plants per time point and two control plants) were inoculated on the abaxial side of leaves as described elsewhere [40] using a high-pressure pumping system to allow the inoculum to penetrate the mesophyll. Control plants were inoculated with sterile saline solution as a mock treatment. The study time course was: 30 mpi, 1, 3 hpi. All 16 samples for mRNA extraction and sequencing were collected separately. Challenged leaf tissues were harvested from each tree and immediately snap frozen in liquid nitrogen. Leaves were stored at -80° C until RNA extraction.

### Total RNA extraction and Illumina sequencing

Total RNA extraction was performed separately for each of the 16 samples using “Plant RNA Reagent” (Life Technologies) large-scale RNA Isolation protocol, according to the manufacturer’s recommendations with the following modifications: all centrifugation steps were carried out at 5,500 x g rather than 2,600 g, and, after total RNA recovery, a final step of aqueous LiCl precipitation was added to reduce carbohydrate carry-over into samples. All samples were aliquoted and stored at -80°C. The purity and concentration of RNA samples were evaluated using a NanoDrop ND-1000 spectrophotometer (Thermo Scientific). Subsequently, samples were sent to the IGA Technology Services (Udine, Italy) for RNA-Sequencing, where they were checked for integrity using an RNA 6000 Nano LABchip Bioanalyzer 2100 (Agilent Technologies, Santa Clara, CA). Briefly, 1.5 μg of total RNA was processed using a ‘TruSeq Stranded mRNA Sample Prep kit’ (Illumina, San Diego, CA) for library preparation. mRNA was purified from each of the 16 samples, enriched using magnetic oligo(dT)-rich beads, and then fragmented using divalent cations at high temperature. Then, cDNA was synthesized, end-filled to produce blunt ends, and an ‘A’ base added to sequence ends. Illumina adapters with index sequences were ligated to the ends of the cDNA fragments. After ligation and purification, samples were amplified by PCR to selectively enrich for cDNA fragments with adapters at both ends. After quality checking of the prepared libraries using a Qubit 2.0 Fluorometer (Invitrogen, Carlsbad, CA) and an Agilent 2100 Bioanalyzer, cDNAs were processed with an Illumina cBot for cluster generation on the flow cell, following the manufacturer’s instructions, and sequenced in single-end mode using a HiSeq2000 sequencing platform (Illumina, San Diego, CA) to generate reads of 50-bp in length.

### RNA-Seq, reads mapping and differential expression analyses

Sequencing reads generated by the sequencing service were imported into CLC Genomics Workbench v.5.0.2 (CLC bio, Aarhus N, Denmark), and filtered using the following parameters: maximum 1 ambiguous nucleotide (N); minimum length 35 nt; maximum length 45 nt; and trimmed for removal of low quality bases at both ends using a quality scores limit of 0.03, and at both ends by 3 nt. To check the selected and trimmed reads before RNA-Seq mapping, a sequencing QC report was created using a built-in function of CLC. The processed reads obtained from each RNA sample were aligned onto the Peach reference genome v1.0 [5] separately, using the CLC RNA-Seq tool, allowing a maximum of two mismatches per read and 10 multiple mapping loci per read; default settings were used for all other parameters. Subsequently, total read counts for each locus were exported from the CLC software and used as an input dataset for the differential gene expression analysis using the DESeq package v.1.12, which employs generalized linear models and offers reliable control of false discoveries by accounting for biological variation [41]. DEGs were inferred separately for each cultivar by direct comparison of total read counts from the two control biological replicates vs. the two biological replicates for each time-point, and computed using default settings according to the instructions of the author of DESeq, with the sequence of commands ‘*estimateSizeFactors*’, ‘*estimateDispersions*’ and ‘*nbinomTest*’. Statistical P value (pval) thresholds were determined using the false discovery rate (FDR) to account for multiple tests of significance (padj, using the Benjamini-Hochberg method). Statistically significant DEGs were defined as those with both padj < 0.05 and log_2_ fold change (FC) > 2 (up-regulated) or < -2 (down-regulated). Venn diagrams were plotted from the DEG lists generated using DESeq using the GeneVenn webtool (genevenn.sourceforge.net).

### GO-term analysis

The Singular Enrichment Analysis (SEA) algorithm was used via the agriGO web tool [42] to analyse Gene Ontology (GO) term enrichment of significantly up-regulated or down-regulated DEGs at each time-point. Briefly, the probe ID number of the best match to an *Arabidopsis* sequence for each peach DEG locus, corresponding to the ‘exclusively regulated genes’ (pre-excluding the commonly regulated IDs from reciprocal lists of up- or down-regulated genes for both cultivars) or to the ‘all regulated genes’ (complete list of all the up- or down-regulated genes), were uploaded in the agriGO tool. The *Arabidopsis* gene model (TAIR9) was used as a reference and the setting parameters were as follows: *‘Hypergeometric’ test*, *‘Yekutieli (FDR under dependency)*’, ‘*Significance level*’: 0.10, ‘*Minimum number of mapping entries*’: 5, ‘*Complete GO*’ ontology type.

### RNA-Seq data validation

To validate the results obtained from bioinformatic and statistical analysis of RNA-Seq, RT-qPCR was performed to analyze four randomly selected genes at each time-point in both cultivars (see section ‘Secondary metabolism’). With this aim, cDNA was synthesized from 1 μg of total RNA using a “Tetro Reverse Transcriptase” kit (Bioline), according to the manufacturer protocol for random hexamers. Primers used in subsequent qPCR reactions were designed from the sequences of the chosen genes (Table A in S1 File). The housekeeping gene used as reference for expression analysis was ppa004776m (Catalase 2); the invariant expression of this housekeeping gene during the time-course experimental conditions was determined from the RNA-Seq bioinformatic analysis in this study and from data published in a previous report of an investigation of the same pathosystem [27]. q-PCR was performed in 96-well plates using the 7500 FAST real-time system (Life Technologies) and a “SensiFAST^™^ SYBR Lo-ROX Kit” (Bioline) in 20 μl reaction volumes according to the manufacturer’s protocol. Before the qPCR expression validation, each primer pair was experimentally checked for the thermodynamic efficiency (serial dilution curve), target specificity, and the absence of any primer dimers (melting curve analysis). The cycling parameters were as follows: 3 min denaturation at 95°C, followed by 40 cycles at 95°C for 10 s, and 60° C for 30 s. Relative expression levels between controls and time points after inoculation were compared using “7500 Software” v.2.0.4 (Life Technologies) according to the 2^-ΔΔCt^ method [43]. The expression levels for each of the 12 targets was tested in all biological replicates of the RNA-Seq experiment using 3 technical replicates.

### Differential exon usage

DEU was inferred using the R package, DEXSeq v.1.8.0 [44], which tests changes in the relative usage of exons caused by the experimental conditions, taking into account biological variability. The whole package comprises two Python scripts and uses generalized linear models of the negative binomial family to identify statistically significant quantitative differences in exon usage. The complete analysis included three main steps. To obtain a GTF file for gene models fully compatible with the format accepted by the first script, a reads mapping session was performed using TopHat followed by the Cufflinks and Cuffmerge tools (Table B in S1 File), on the Galaxy open source web-based platform [45]. Cuffmerge output GTF file and TopHat output BAM alignment files were exported and used as input for the first and the second Python scripts, respectively. Subsequently, the count files produced by the second script, corresponding to each biological replicate, were imported in DEXSeq and used to create the ‘*ExonCountSet*’, including the biological replicates and the treatments to be compared (i.e., control vs. timepoint). Subsequently, the ‘*size factors*’ for the normalization between samples, due to different depths of sequencing were calculated. Next, dispersion between the biological replicates was estimated to determine the variability of the data. Finally, an ‘*ExonCountSet*’, including dispersion estimates and the ‘*size factors*’, was used to infer DEU between control and treatments (time points). The significance of the inferences was tested by controlling both the FDR through Benjamini-Hochberg method (padj < 0.05) and setting the threshold at an FC > 2 or < -2.

## Results and discussion

### Illumina sequencing and RNA-Seq mapping

To explore the very early defense response of resistant (‘Redkist’) and susceptible (‘JH Hale’) peach cultivars to Xap, the transcriptomes of infected leaves at 30 mpi, 1, and 3 hpi were subjected to massively parallel sequencing using the Illumina NGS platform. To facilitate robust biological interpretation, RNA-Seq experimental designs should include independent biological replicates [46–48]; therefore, we included two independently grafted trees as biological replicates for each analysis time point along with controls. The use of two biological replicates reduces the power of statistical analyses [47,48]; therefore this study cannot draw strong conclusions; however, it is a useful exploration that facilitates hypothesis generation [41,49,50].

Each biological sample was extracted, sequenced, and aligned to the peach genome (Peach v1.0) separately; neither RNA samples nor sequence reads were pooled. After quality selection and trimming, an average of 68 million single reads were available for mapping per sample, which together (1.09 billion reads) provided an extensive snapshot of the defense response of peach to Xap (Table C, D, E in S1 File). The average quality score per base was 39.1, with an N base (ambiguous nucleotide) frequency of 0.01% (Table 1). Reads were aligned on the Peach v1.0 reference genome [5] using the CLC-bio software; on average 91.8% of available reads were mapped onto the genome (Table 2).

**Table 1:** RNA-Seq read statistics before mapping and after quality selection and trimming.

			Control	30 mpi	1 hpi	3 hpi
**‘Redkist’**	Replicate 1	N. reads	65,051,736	69,463,949	64,229,441	87,856,453
		% GC	45.31	44.62	45.14	44.42
		Q score/base	39.78	39.27	38.80	39.22
		N %	0.01	0.01	0.01	0.01
	Replicate 2	N. reads	64,527,025	56,843,107	55,679,435	62,133,913
		% GC	45.71	44.58	44.70	44.51
		Q score/base	39.73	38.87	38.62	38.82
		N %	0.01	0.01	0.01	0.01
**‘JH Hale’**	Replicate 1	N. reads	82,337,544	65,869,363	61,879,157	82,155,732
		% GC	45.24	44.88	44.58	44.82
		Q score/base	39.24	38.84	39.22	38.80
		N %	0.01	0.01	0.01	0.01
	Replicate 2	N. reads	64,613,903	57,009,995	84,416,784	64,217,392
		% GC	45.04	44.98	44.37	45.05
		Q score/base	39.22	38.84	39.24	38.78
		N %	0.01	0.01	0.01	0.01

Reads obtained by deep sequencing of leaf samples of two peach cultivars ‘JH Hale’ (susceptible) and ‘Redkist’ (resistant) at different time points after Xap inoculation (30 mpi, 1 and 3 hpi). **% GC**, relative GC-content (%); **Q score/base**, mean PHRED quality score (Q score) per nucleotide; **N %**: relative percentage of ambiguous nucleotides.

**Table 2:** RNA-Seq statistics for reads mapped to the peach genome. Data are presented as averages of two biological replicates.

		Control	30 mpi	1 hpi	3 hpi
**‘Redkist’**	Average n. of mapped reads	56,689,826	59,326,511	55,823,367	70,837,711
	Average % of mapped reads	87.49	93.91	93.13	94.36
	Average n. of unmapped reads	8,099,555	3,827,017	4,131,116	4,157,472
	Average % of unmapped reads	12.51	6.09	6.87	5.64
	Average n. of exon reads	43,894,705	46,658,322	44,364,560	56,476,587
	Average % of exon reads	77.42	78.65	79.46	79.60
	Average n. of intron reads	12,795,121	12,668,190	11,458,807	14,361,125
	Average % of intron reads	22.58	21.35	20.54	20.40
**‘JH Hale’**	Average n. of mapped reads	65,439,159	56,194,189	67,786,353	67,453,001
	Average % of mapped reads	89.11	91.45	92.57	92.03
	Average n. of unmapped reads	8,036,565	5,245,491	5,361,618	5,733,562
	Average % of unmapped reads	10.89	8.55	7.43	7.97
	Average n. of exon reads	50,332,826	43,857,044	52,703,130	52,878,320
	Average % of exon reads	76.94	78.09	77.76	78.35
	Average n. of intron reads	15,106,334	12,337,272	15,083,223	14,574,681
	Average % of intron reads	23.06	21.91	22.24	21.65

**Fig 1:**
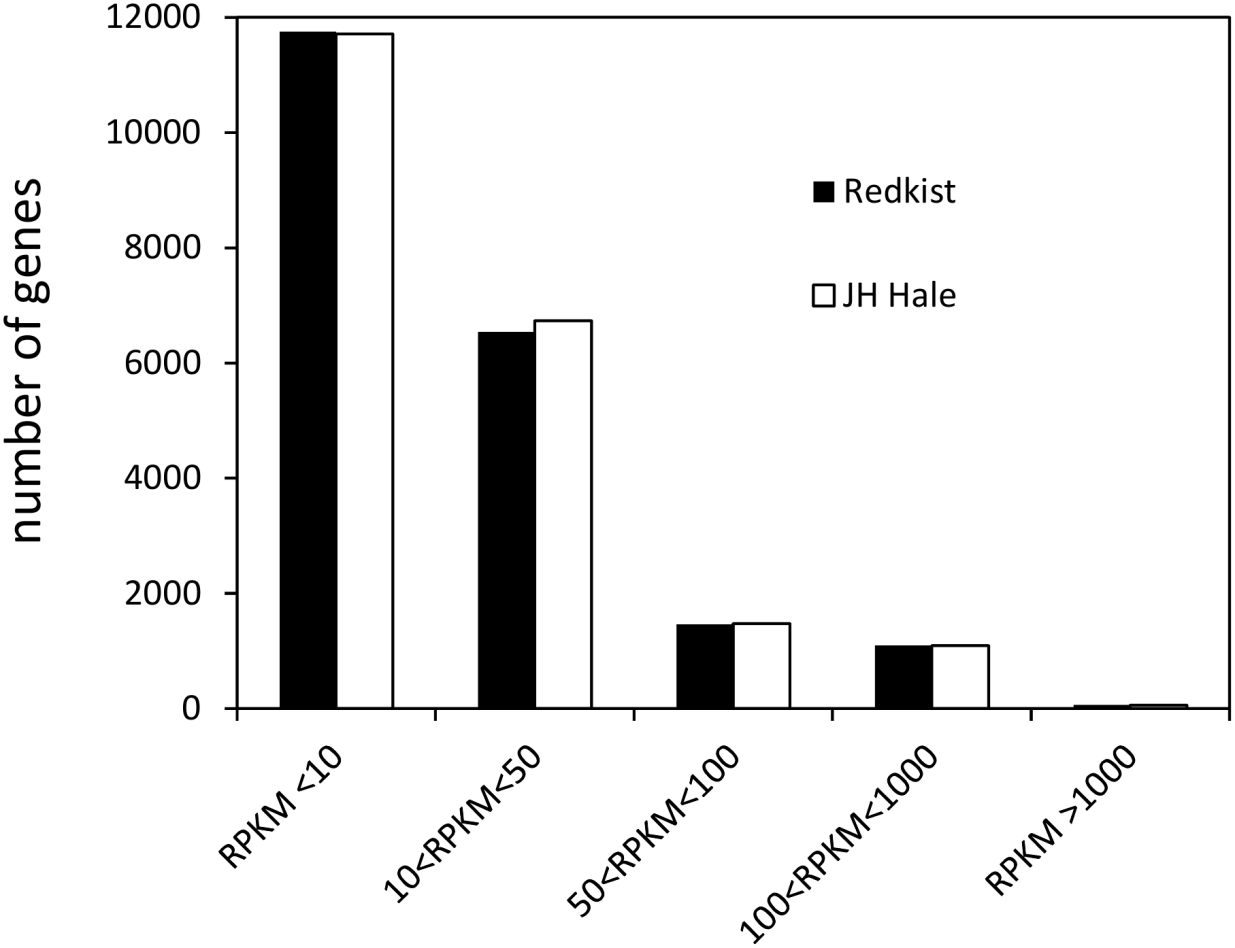
Distribution of frequency classes of gene expression levels (RPKM) after Xap inoculation on leaves of ‘JH Hale’ (susceptible) and ‘Redkist’ (resistant) peach cultivars.

Reads per kilobase per million mapped reads (RPKM), as proposed by Mortazavi [51], estimates the gene expression level of a gene normalized for both transcript length and library sequencing depth, allowing a direct comparisons of expression levels within and between samples [51]. During the time course, we detected the expression of 20,837 genes corresponding to 75% of predicted genes in the Peach v1.0 reference sequence. The minimal expression detected was 0 and the maximum value was 12,631.62 RPKM for the metallothionein gene, ppa014506m, in ‘Redkist’. Analysis of expression level category distribution clearly demonstrated that more than half of genes (55.9%) were expressed at RPKM levels < 10, with 87.5% expressed at RPKM levels < 50 (Fig 1).

### Differential expression: Activation kinetics and intensity

**Fig 2:**
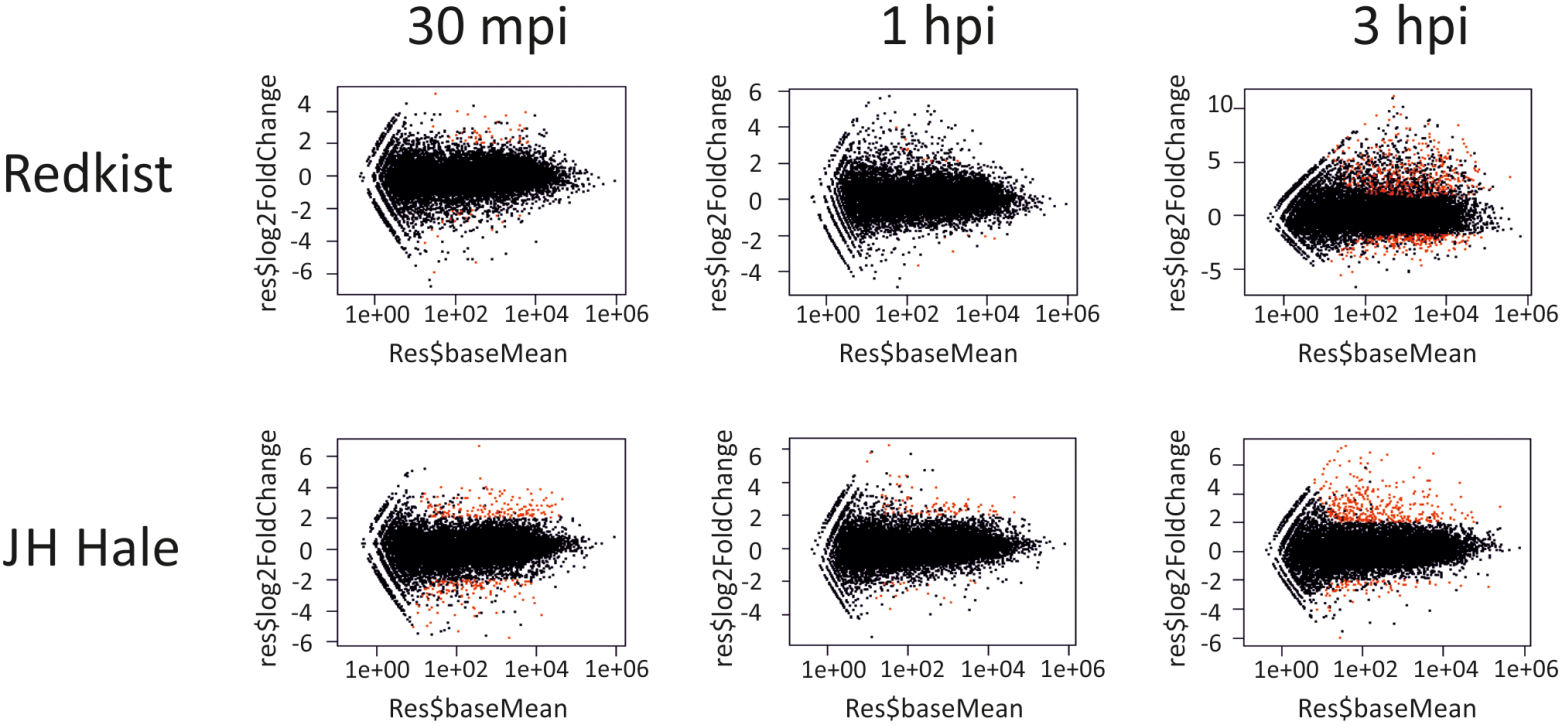
MA plot of genes differentially expressed (red dots) by two peach varieties during a time course after Xap infection. DEGs were selected by filtering based on log_2_ (FC) > 2, or log_2_ (FC) < -2, and FDR < 0.05.

Before performing the differential expression analyses, R^2^ correlations (range 0.91–0.98) confirmed the similarity between the biological replicates (S1 Fig). Analysis of DEGs revealed the very early activation kinetics and global numbers of genes regulated in the peach defense response to Xap. Interestingly, both cultivars showed similar activation kinetics, reacting as early as 30 mpi re-modulating gene expression, then showing a significantly reduced response at 1 hpi, followed by a much wider reaction, in terms of the number of regulated genes, at 3 hpi (Fig 2). Surprisingly, the susceptible cultivar modulated (up-regulated and down-regulated) a higher number of genes across the whole time-course (821) than the resistant cultivar (714); moreover, at the two earliest time-points (30 mpi and 1 hpi) ‘JH Hale’ exhibited altered expression of 285 and 99 genes, respectively, whereas ‘Redkist’ re-modulated expression of only 67 and 16 genes, respectively (Table 3 and Table E in S1 File). In contrast, at 3 hpi the resistant cultivar exhibited a massive scale reprogramming of 631 genes, representing the 88% of all the genes regulated across the entire time course, 488 of which were resistant specific (S2 Fig). This differed markedly to the 437 genes with altered expression in ‘JH Hale’, which represented 53% of all regulated genes (Table 3 and Table E in S1 File), 294 of which were specifically regulated in the susceptible cultivar (S2 Fig). Taken together, these data regarding DEGs reveal an interesting picture of the kinetics in response to Xap, with an activation/drop/reaction (at 30 mpi, 1, and 3 hpi, respectively) pattern observed in both cultivars, with a broader response in the susceptible cultivar up to 1 hpi, which was exceeded at 3 hpi by the response of the resistant plant (Fig 2 and Table 3). These kinetics confirm in peach findings generated using the model *Arabidopsis*, after chitin elicitation [22] and *Pseudomonas syringae* pv. *tomato* infiltration [21]. Moreover, the data from different time points in peach confirmed that variation in resistance and susceptibility to Xap is not determined by the total number of regulated genes, but is presumably determined via expression kinetics and intensity of induction [28].

**Table 3:** Transcriptome remodeling in terms of numbers of regulated genes in ‘Redkist’ (resistant) and ‘JH Hale’ (susceptible) cultivars during a time course (30 mpi, 1, and 3 hpi) after Xap infection.

	‘Redkist’			‘JH Hale’		
	30 mpi	1 hpi	3 hpi	30 mpi	1 hpi	3 hpi
n. up-regulated genes	43	10	445	160	80	364
n. down-regulated genes	24	6	186	125	19	73
total n. regulated genes	67	16	631	285	99	437
average fold change up-regulated genes	7.2	6.8	37.5	7.3	8.3	13.2
average fold change down-regulated genes	-10.7	-6.3	-7.1	-8.2	-6.2	-6.9
n. genes differential exon usage	205	33	489	182	31	401

Further differences between the responses of the cultivars increased after filtering for highly up-regulated genes (FC > 100) at 3 hpi. ‘Redkist’ strongly up-regulated 31 genes (Table 4), whose FC ranged from 101.4 (ppa004445m) to a maximum of 2,215.8 (ppa026872m), the majority of which had putative roles in stress and pathogen resistance. In contrast, six genes were strongly up-regulated by ‘JH Hale’ at 3 hpi (Table 4) and the gene exhibiting the maximum fold change (152.8) was a DNA topoisomerase 1 beta (ppa019677m). These data regarding highly up-regulated genes prompted us to calculate the mean-FC of up-regulated and down-regulated genes for each time point in both the cultivars, confirming that ‘Redkist’ reacted more strongly at 3 hpi than ‘JH Hale’ in up-regulating gene expression (average FC values: 37.5 and 13.2, respectively) with the average FC 2.8 times higher in resistant than that in susceptible plants (Table 3).

**Table 4:** Highly up-regulated genes (fold change > 100) at 3 hpi with Xap in ‘Redkist’ (resistant) and ‘JH Hale’ (susceptible) peach cultivars.

**‘Redkist’**			
**Gene ID**	**Gene symbol**	**Function**	**Fold change**
ppa004445m	*ATTT12*; *TT12*	MATE efflux family protein	101.4
ppa004569m	*CYP82G1*	Cytochrome P450, family 82, subfamily G	101.5
ppa009384m		Pathogenesis-related thaumatin superfamily protein	105.7
ppa021326m	*CYP79B3*	Cytochrome P450, family 79, subfamily B	106.9
ppa018972m	*ACA4*	Autoinhibited Ca(2+)-ATPase, isoform 4	110.6
ppa005318m	No acronym	HXXXD-type acyl-transferase family protein	111
ppa023986m		S-adenosyl-L-methionine-dependent methyltransferases	124.8
ppa010831m	*ERD9*	Glutathione S-transferase family protein	125
ppa020145m		RNA 3'-phosphate cyclase/enolpyruvate transferase	125.4
ppa009630m	*PIP2*; *5*,*PIP2D*	Plasma membrane intrinsic protein 2;5	125.5
ppa004095m	*CYP82G1*	Cytochrome P450, family 82, subfamily G	126.7
ppa010729m		Pathogenesis-related protein	127.6
ppa006485m	*MAPKKK15*	Mitogen-activated protein kinase kinase kinase 15	131.8
ppa003380m	*TPS14*	Terpene synthase 14	137.1
ppa007615m	*ELI3-1*	Elicitor-activated gene 3–1	137.4
ppa004774m	*ACS1*	ACC synthase 1	146
ppa012865m		RING/U-box superfamily protein	154.4
ppa018623m		Pathogenesis-related protein	159.9
ppa016292m	*TPS03*	Terpene synthase 03	187.4
ppa017145m	*LWD1*	Transducin/WD40 repeat-like superfamily protein	197.1
ppa001216m	*ATLOX1*; *LOX1*	Lipoxygenase 1	201
ppa021232m	*ELI3-1*	Elicitor-activated gene 3–1	269.7
ppa013491m		No annotation	286
ppa023341m	*TPS03*	Terpene synthase 03	340.4
ppa007004m	*CHY1*	Beta-hydroxyisobutyryl-CoA hydrolase 1	354.3
ppa010133m		NAD(P)-binding Rossmann-fold superfamily protein	355.4
ppa015643m	*CBF2*	C-repeat/DRE binding factor 2	477.9
ppa025605m	*UGT85A7*	UDP-glucosyl transferase 85A7	656.8
ppa012414m	*WRKY75*	WRKY DNA-binding protein 75	706.7
ppa007627m	*ELI3-1*	Elicitor-activated gene 3–1	1,100.7
ppa026872m	*PLA2A*	Phospholipase A 2A	2,215.8
**JH Hale**			
**Gene ID**	**Gene symbol**	**Function**	**Fold change**
ppa021326m	*CYP79B3*	Cytochrome P450, family 79, subfamily B	103
ppa023341m	*TPS03*	Terpene synthase 03	107.3
ppa007627m	*ELI3-1*	Elicitor-activated gene 3–1	116
ppa007488m	*OPR2*	12-oxophytodienoate reductase 2	118.7
ppa015919m	*BG5*	Beta-1,3-glucanase 5	138
ppa019677m	*TOP1BETA*	DNA topoisomerase 1 beta	152.8

**Fig 3:**
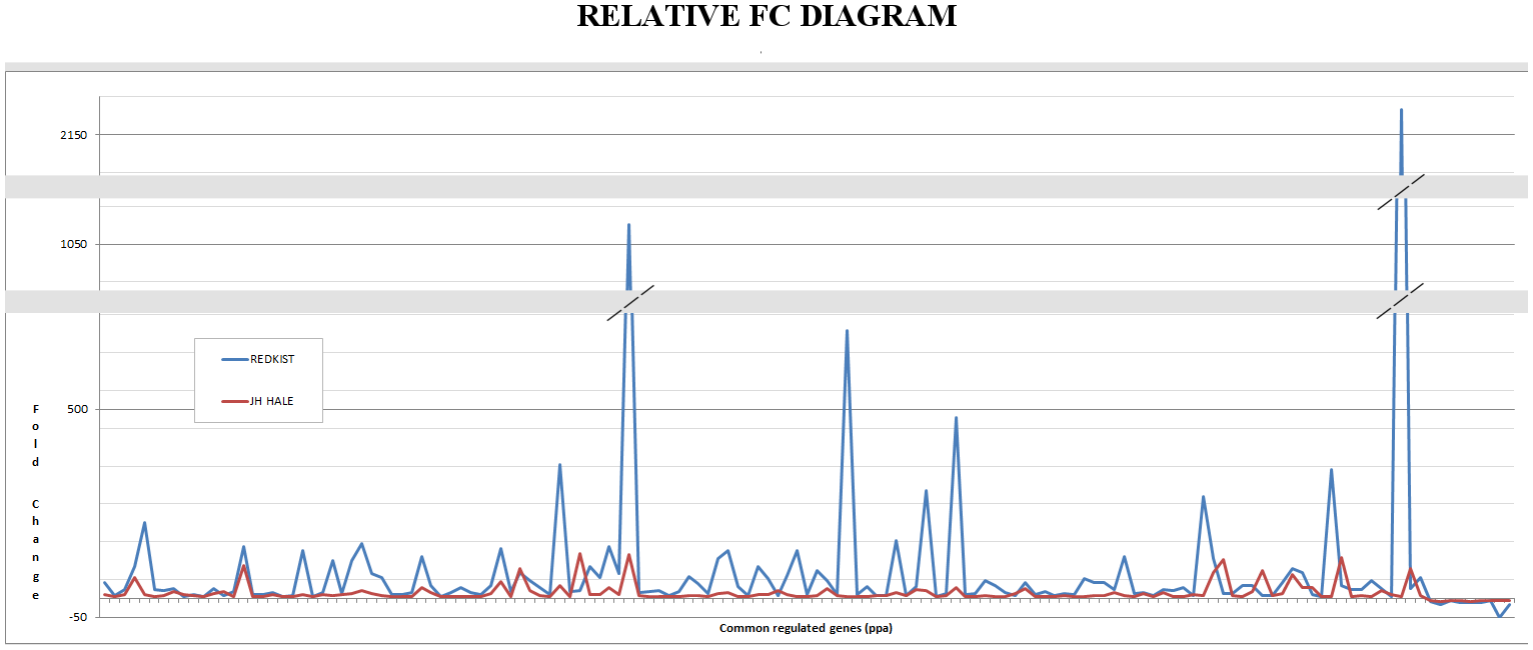
Relative fold change (FC) of expression levels of genes regulated in both ‘Redkist’ (resistant) and in ‘JH Hale’ (susceptible) peach cultivars after Xap infection; max FC = 2216, min FC = -49.

The overlap in DEGs between the two genotypes was plotted in a Venn diagram (S2 Fig) and the relative list of the 134 regulated genes by both genotypes at 3 hpi was used to plot a relative FC diagram (Fig 3). The plot illustrates that ‘Redkist’ displayed a higher FC for the majority of up-regulated genes in common between the two cultivars, confirming that, not only that the global average FC was higher in ‘Redkist’ at 3 hpi, but it was also higher in genes up-regulated by both cultivars.

The results of bioinformatic analyses of RNA-Seq data were validated by RT-qPCR (Table F in S1 File). which demonstrated a high degree of correlation (R^2^ = 0.98) between the two approaches, supporting the relevance of the RNA-Seq statistical analyses.

### Functional classification of DEGs

To achieve a deeper knowledge of DEGs in peach responses to Xap, we performed GO-term singular enrichment analysis (SEA) of all sets of genes differentially up- and down-regulated at 30 mpi, 1, and 3 hpi in each cultivar using the AgriGO tool. Table G in S1 File includes lists of significant GO-term categories for each time point, according to the following categories: biological process (P), molecular function (F), and cellular component (C). In ‘Redkist’, GO analysis detected 33, 3, and 131 categories among ‘all up-regulated genes’ (complete list of all up-regulated genes) at 30 mpi, 1, and 3hpi, respectively; whereas among down-regulated genes 4 and 40 categories were identified at 30 mpi and 3 hpi, and there were no categories significantly down-regulated at 1 hpi. In ‘JH Hale’ 52, 14, 113 categories were up-regulated with 33, none, and 5 down-regulated at 30 mpi, 1, and 3 hpi. These results confirmed the results of DEG analysis, indicating that the susceptible cultivar is more reactive and initiates a more complex biological response at 30 mpi and 1 hpi, whereas at 3 hpi a more pronounced response was observed in the resistant cultivar. Across the whole time-course, the lists of DEGs for both cultivars (Table G in S1 File) were enriched for terms suggesting a vast transcriptional reprogramming of the signal transduction system, transcription machinery, primary and secondary metabolism, redox balance, stress response, and immune system. In summary, both cultivars activated a defense response, which relied on similar biological processes and cellular components; however, the timing of this response differed between them.

At 30 mpi, only ‘JH Hale’ ‘exclusively up-regulated’ (pre-excluding the commonly regulated IDs from the reciprocal lists for both cultivars) genes characterized by the GO-terms in the biological processes (P): ‘defense response’, ‘immune response’, ‘immune system process’, ‘response to other organism’, or ‘innate immune response’ and among the molecular functions (F) ‘transmembrane receptor activity’, receptor activity’, ‘molecular transducer activity’, ‘signal transducer activity’, ‘kinase activity’, and ‘protein kinase activity’ (Table 5). Moreover, at this time point only ‘JH Hale’ exclusively down-regulated genes corresponding with significant enrichment for the terms: ‘regulation of transcription’, ‘transcription’, and ‘regulation of gene expression’ among biological processes (P); and ‘transcription regulator activity’, ‘transcription activator activity’, ‘transcription factor activity’, and ‘DNA binding’ among the molecular functions (F). These results of analyses of the subset of genes exclusively down-regulated by the susceptible cultivar at 30 mpi suggest a very early attempt to respond to the infection, but with the limitation of restricting the expression of genes involved in the transcriptional activity, which is crucial for a successful resistance response.

**Table 5:** Significantly enriched GO terms (Biological processes) in the susceptible cultivar ‘JH Hale’ at 30 mpi with Xap.

Up-regulated genes			Down-regulated genes		
Term	Number	FDR	Term	Number	FDR
binding	48	0.008	biological regulation	23	0.023
cellular localization	6	0.083	cell wall	6	0.024
defense response	10	0.0028	DNA binding	17	0.094
establishment of localization	13	0.036	endopeptidase activity	5	0.033
establishment of localization in cell	6	0.06	external encapsulating structure	6	0.024
immune response	6	0.018	plant-type cell wall	6	0.0062
immune system process	6	0.018	plasma membrane	11	0.046
innate immune response	5	0.055	regulation of biosynthetic process	15	0.009
kinase activity	11	0.064	regulation of cellular biosynthetic process	15	0.009
localization	13	0.046	regulation of cellular metabolic process	15	0.013
membrane	22	0.077	regulation of gene expression	15	0.012
molecular transducer activity	6	0.024	regulation of macromolecule biosynthetic process	15	0.009
multi-organism process	10	0.0028	regulation of macromolecule metabolic process	15	0.014
phosphotransferase activity, alcohol group as acceptor	9	0.064	regulation of metabolic process	15	0.023
plasma membrane	11	0.077	regulation of nitrogen compound metabolic process	15	0.009
protein binding	24	0.00011	regulation of nucleobase, nucleoside, nucleotide and nucleic acid metabolic process	15	0.009
protein kinase activity	8	0.064	regulation of primary metabolic process	15	0.01
receptor activity	5	0.01	regulation of transcription	15	0.009
response to biotic stimulus	10	0.00092	response to abiotic stimulus	13	0.009
response to other organism	10	0.00092	response to carbohydrate stimulus	5	0.013
response to stimulus	27	0.00092	response to chemical stimulus	17	0.0061
response to stress	17	0.0072	response to chitin	5	0.0061
signal transducer activity	6	0.024	response to endogenous stimulus	10	0.017
transcription factor activity	14	0.041	response to external stimulus	6	0.023
transcription regulator activity	14	0.066	response to hormone stimulus	9	0.029
transmembrane receptor activity	5	0.0077	response to jasmonic acid stimulus	5	0.01
transport	13	0.036	response to light stimulus	6	0.09
vesicle-mediated transport	5	0.028	response to organic substance	15	0.0013
			response to osmotic stress	5	0.081
			response to radiation	6	0.099
			response to salt stress	5	0.054
			response to stimulus	28	0.0013
			response to stress	15	0.034
			response to wounding	6	0.0029
			transcription	15	0.01
			transcription factor activity	16	0.025
			transcription regulator activity	19	0.0059

Lists of GO-terms (biological processes) of genes ‘exclusively up-regulated’ and ‘exclusively down-regulated’ at 30 mpi only in the susceptible cultivar, ‘JH Hale’.

Strikingly, at 3 hpi only ‘Redkist’ reacted by specifically down-regulating genes in the biological processes categories (P) ‘photosynthesis’, ‘photosynthesis, light reaction’ (Table H in S1 File). Similar behavior was also reported by the clone, GF305, in response to Xap inoculation, whith down-regulation of a set of 19 photosynthesis-related genes after 12 hpi [27]. Furthermore, similar results were also described in an analysis of the the transcriptomes of resistant soybean plants inoculated with *Pseudomonas syringae* pv. *glycinea* with 94 chloroplast-related gene transcripts significantly down-regulated specifically in the resistant strain at 8 hpi [52]. Overall, our data fit with previous findings, supporting a key role for the down-regulation of photosynthesis-related genes that may be crucial to the response of the resistant genotype to Xap in the very early phase of infection [53,54].

### Receptors and signal transduction

Cysteine-rich RLK (CRK) genes are a large family, with 40 members in *Arabidopsis*, some of which are involved in plant defense and development, and up-regulated after bacterial infection, or salicylic acid (SA) or H_2_O_2_ treatment [55,56]. A group of them (Table I in S1 File) were differentially up-regulated in ‘Redkist’ and ‘JH Hale’ cultivars at 3 hpi. The *CRK2*-like (ppa022817m) and *CRK25*-like (ppa026781m) were up-regulated only by ‘Redkist’ plants, whereas levels of the *CRK42*-like gene (ppa002530) were increased in both cultivars with a similar FC value (10.2 and 6.6, respectively). The *CRK2*-like gene (ppa022817m) maps 1.2 Mb from the closest marker (SNP_IGA_295433, Scaffold_3:1,977,321, Peach v1.0) for a QTL for Xap resistance on linkage group 3 (Xap.Pp.OC-3.1) identified by Yang et al. [17].

Resistance genes belonging to the family NBS-LRRs represent an intracellular arm of the perception system which allows plants to sense pathogen effector activity and trigger strong defense reactions (effector triggered immunity-ETI) which often culminate in the hypersensitive response (HR) [57,58]. Peach NBS-LRRs-like genes were up-regulated throughout the time-course (30 mpi, 1, and 3 hpi) in both ‘Redkist’ (4, not significant, and 5 genes respectively) and ‘JH Hale’ (20, 4, and 5 genes, respectively) (Table I in S1 File). These unexpected findings, indicating that the susceptible cultivar up-regulates a greater number of NBS-LRR genes than the resistant one, highlight an apparent paradox, in that the response of the susceptible plants appears to be more robust than that of the resistant cultivar.

During the time-course a number of signal transduction-like protein encoding genes exhibited differential regulation, including those encoding the following proteins: protein kinase, calcium dependent protein kinase, calmodulin-like protein, calmodulin-like binding protein, MAP kinase kinase kinase, leucine rich repeats protein, serine/threonine kinase, and transducin/WD-40 like protein. Across the entire time course, ‘JH Hale’ plants up-regulated significantly more genes than the resistant cultivar (43 vs. 30) and these variations also exhibited differences in kinetics (Table I in S1 File). At 30 mpi and 1 hpi ‘JH Hale’ increased the expression of 32 genes, whereas only 5 were increased in ‘Redkist’, in contrast, at 3 hpi, expression of 25 genes was enhanced in ‘Redkist’, with only 11 up-regulated in ‘JH Hale’. Our data regarding DEGs involved in signaling events confirm previously reported findings indicating differences in the kinetics of DEG expression. Namely, the susceptible cultivar was already exhibiting a response to infection at 30 mpi by modulating the signal transduction machinery; however, at 3 hpi the resistant cultivar began to adapt its signaling pathways by more than doubling the number of up-regulated genes involved in signaling events.

Reactive oxygen species (ROS) are a key component of the signaling events involved in plant development and stress responses [59]. Different enzymes responsible for of ROS production in the plant cell have been described [60–64]. Recently, a direct role for amine oxidases has also been demonstrated in plant resistance and in the HR through ROS production via polyamine oxidation, indicating that these enzymes contribute to both to the innate and inducible defense responses to biotic stress [65,66]. At 3 hpi ‘Redkist’ up-regulated three genes encoding proteins with amine-oxidase activity, whereas ‘JH Hale’ up-regulated only one. Only the resistant cultivar up-regulated a set of genes encoding a Copper amine oxidase (CuAO)-like (ppa016301m) (FC: 24.7), and a Poly-amine oxidase (PAO) 2-like (ppa005584m) (FC: 6.4) protein, while both cultivars up-regulated one *PAO 1*-like gene (ppa004511m) (resistant FC, 12.9 and susceptible FC, 11.6 the susceptible) (Table E in S1 File). Our data are in agreement with previous studies of other pathosystems which showed that increased CuAO or PAO expression levels and their relative enzymatic activity were positively correlated with HR and resistance in *Nicotiana* [67,68]. Also, in *Citrus sinensis* a significantly higher PAO enzymatic activity resulted in a more evident HR, increased accumulation of H_2_O_2_, and reduced susceptibility to *Xanthomonas axonopodis* pv. *citri* artificial infection [69]. Furthermore, levels of both CRK and CAO genes are correlated with HR during infection [55,56,67]. Finally, the *CuAO*-like gene (ppa016301m) maps 1 Mb from the closest marker (SNP_IGA_5891 at scaffold_1:1,957,063) associated with a QTL (Xap.Pp.OC-1.1) in linkage group 1 [17].

### Transcription factors

Transcription factors (TFs) are the terminal targets of signaling events downstream of pathogen perception. Even during the very early phases of interaction, both cultivars modulated (up-regulation and down-regulation) the expression of a total of 50 TFs (Table J in S1 File). One difference between the responses of the two cultivars was the number of TFs with altered expression levels across the entire time course: 46 for the susceptible and 26 for the resistant cultivar. This difference was not a reflection of up-regulated genes, where there was a similar total number of genes and the same kinetics in each cultivar (‘Redkist’, 3, 0, and 13; ‘JH Hale’, 7, 0, and 14 genes up-regulated at 30 mpi, 1, and 3 hpi, respectively), rather it is caused by the down-regulation of 18 putative TFs at 30 mpi in ‘JH Hale’ (‘JH Hale’: 18, 2, and 5; ‘Redkist’: 3, 1, and 6 genes down-regulated at 30 mpi, 1, and 3 hpi) as also determined by the GO analysis. A further difference between the resistant and the susceptible responses was the expression of WRKY-like TFs: a gene family comprising 74 members in *Arabidopsis* [70] and 58 putative homologs in *P*. *persica* [71] whose encoded proteins are well known for their role in stress responses [70,72]. At 3 hpi, only ‘Redkist’ up-regulated the TF *WRKY28*-like gene (ppa024027m, FC 7.4) and in agreement with the observations in *Arabidopsis* that the gene *AtWRKY28* is up-regulated in response to the flagellin mimetic peptide, flg22 [23] and by *P*. *syringae* leaf infiltration [73,74]. Furthermore, *AtWRKY28* is a transcriptional activator of the isochorismate synthase the key enzyme in one of the two pathways for biosynthesis of SA [75,76], a crucial molecule in plant resistance. Also at 3 hpi, both cultivars increased expression levels of the *WRKY75*-like gene (ppa012414m); however, there was a dramatic difference between them in the FC (‘Redkist’, 706.7; ‘JH Hale’, 4.6). *AtWRKY75* is associated with abiotic resistance [70]; however, Thilmony et al. reported its up-regulation in response to *Pseudomonas syringae* pv. *tomato* DC3000 infection in *Arabidopsis* [77]. A more recent study in strawberry (*Fragaria* × *ananassa*), which, like peach, is a Rosaceous species, provided evidence of its involvement in biotic stress resistance. The overexpression of the *Fragaria FaWRKY1*, a homolog of *AtWRKY75*, in an *Arabidopsis WRKY75*-defective mutant, was associated with resistance to *Pseudomonas syringae* pv. *tomato*, with strong induction of oxidative burst generation and glutathione-S-transferase (GST) activity [78]. The peach *WRKY75* homolog, ppa012414 (on scaffold_5:13,581,887..13,583,455, Peach v1.0), was highly up-regulated in the resistant cultivar (FC 706.4), and maps within the 1 LOD confidence interval containing a major QTL for resistance to Xap in apricot that accounts for 53% of the phenotypic variation [27], at 172 kb from the QTL peak at marker UDAp-452 (Scaffold_5 13755609 bp in Peach v1.0). The same gene is 1.6 Mb from a major QTL for resistance to Xap (Xap.Pp.OC-5.1; closest maker SNP_IGA_594090, Scaffold_5: 11,952,452, Peach v1.0) mapping to the middle part of LG5 in peach [17]. Together, this evidence supports a key role for the peach *WRKY75*-like gene (ppa12414m) in Xap resistance during the early phase of the infective process.

### Secondary metabolism

Our RNA-Seq analysis detected a number of up-regulated genes involved in biosynthesis of secondary metabolites, mainly at 3 hpi, when 69 genes were up-regulated in ‘Redkist’ and 48 in ‘JH Hale’ (Table K in S1 File). Some of these genes encode enzymes that control the phenyl-propanoid ‘core pathway’ (*PAL*, *C4H*, *4CL*), and downstream flavonoids, stilbens, lignins, and genes for terpens biosynthesis. Our data show that only ‘Redkist’ plants increased their expression levels of all three genes encoding enzymes of the phenyl-propanoid ‘core pathway’ and those of two different homologous genes, annotated as *PAL*-like (ppa002328m, ppa002099m), with FC 17 and 26.7 respectively; whereas, ‘JH Hale’ up-regulated only the latter with a lower FC (19.2). *PAL* is the first step and the master regulator of the phenyl-propanoid pathway; it has previously been reported as up-regulated in response to biotic and abiotic stress [79], and has an important role in *Xanthomonas* resistance. Specifically, Kim and Hwang demonstrated by silencing or overexpressing the *PAL1* gene that its expression levels are crucial in pepper for resistance to *Xanthomonas campestris* pv. *vesicatoria* [80].

Furthermore, ‘Redkist’ exhibited an average FC, calculated from up-regulated genes involved in secondary metabolism, of more than double that of ‘JH Hale’ (45.7 vs. 19.5, respectively), indicating that, for peach, the intensity of induction is also a key difference between resistance and susceptibility, as previously demonstrated for the responses of *Arabidopsis* to both elicitors and to bacterial pathogens [22,73,81].

### Differential exon usage: A preliminary analysis

DEU is a eukaryotic molecular mechanism that functions in a range of physiological processes in response to developmental stimuli or environmental challenges [82]. It has also been described in many plant-pathogen interactions and proposed as a strategy to modulate a rapid and transient response to pathogen attack at the mRNA level [83–85]. To characterize the peach defense reaction to Xap at the exon level, we performed DEU analysis, using the RNA-Seq data. Similarly to analysis of DEGs, the results indicated that both cultivars had already begun to remodel their transcriptomes at 30 mpi. In ‘Redkist’ this mechanism involved 727 genes across the whole time course, whereas in ‘JH Hale’ 614 underwent DEU. Moreover, the resistant cultivar exhibited differential usage of exons in a larger number of genes at every time point compared with the susceptible cultivar (205, 33, and 489 vs. 182, 31, and 401 genes at time points 30 mpi, 1, and 3 hpi, respectively; Table 3 and Table L in S1 File). It is interesting to note that, at 30 mpi and 1 hpi, ‘Redkist’ reacted to the infection by undergoing DEU in a markedly higher number of genes than there were DEGs at the same time points (205 and 33 vs. 67 and 16 at 30 mpi and 1 hpi, respectively), which was opposite to the behavior of ‘JH Hale’ in which the number of DEGs was significantly higher than the number of DEUs (182 and 31 vs. 285 and 99 at 30 mpi and 1hpi, respectively) (Table 3). Interestingly, in the resistant strain the number of DEUs at 30 mpi and 1 hpi was greater than the number of DEGs, whereas at 3 hpi the opposite was the case (Table 3). To our knowledge this is the first report of DEU as a mechanism of transcriptome re-modelling in the peach resistance response to a bacterial pathogen.

Several studies, reviewed by Yang et al. [83], have noted DEU in a number of NBS-LRR resistance gene mRNAs after recognition of their cognate effector, and DEU is key to the development of complete resistance. Interestingly, DEU analysis detected a sub-set of seven *TIR-NBS-LRR* (*TNL*) genes at 3 hpi only in ‘Redkist’ (Table L in S1 File). *TNL* genes are a major class of resistance (*R*) genes contributing to ETI in dicotyledonous plants. Five *TNL* genes (ppa017983m, ppa018951m, ppa019721m, ppa025848m, ppa026289m) exhibited DEU after infection, and cluster in a 120 kb region on chromosome 1 (12,421,233–12,543,473 nt), located at 400–500 kb from a major QTL (Xap.Pp.OC-1.2, closest markers SNP_IGA_34306 at 12,014,979 nt and SNP_IGA_39717 at 12,919,375, Peach v1.0) for Xap resistance in peach [17].

### Conclusions

Transcriptome analysis represents a powerful tool for the identification of genes putatively involved in the control of important agronomic traits, such as disease resistance. This study broadens the knowledge of the very early defense responses of resistant (‘Redkist’) and susceptible (‘JH Hale’) peach cultivars to an *Xanthomonas arboricola* pv. *pruni* attack by RNA-Seq analysis. A set of genes differentially reprogrammed after Xap infection was identified. The functions of the proteins encoded by these genes was only determined *in silico* via sequence homology with the *Arabidopsis thaliana* genome and, as such, must be considered putative. Both cultivars counteracted the infection by up-regulating genes involved in perception, signal transduction, transcription, and secondary metabolism. The responses of the two genotypes revealed an apparent paradox: the susceptible cultivar up-regulated the expression of a larger number of genes during the overall time course (821 vs. 714), as well as at time points 30 mpi (285 vs. 67) and 1 (99 vs. 16) hpi, suggesting a more robust response to the pathogen. In contrast, at 3 hpi the resistant cultivar was much stronger in terms of quantitative induction and number of regulated genes (631 vs. 337). Furthermore, our study highlights a set of candidates, mapping to QTLs for resistance to Xap and regulated only in the resistant cultivar, including WRKY-like TFs and a cluster of TNL-like, CRK-like, and CuAO-like proteins. These reepresent a promising starting point for future investigations aimed at understanding the roles of these proteins in resistance to Xap and pave the way for their future use in marker assisted breeding or gene editing and cis-genesis applications.

## Supporting information

S1 Fig Pairwise scatter plots of biological replicates demonstrating the high degree of correlation between replicates in all experimental conditions. (PPTX)

S2 Fig Venn diagrams of up- and down-regulated DEGs in ‘Redkist’ and ‘JH Hale’ in response to Xap infection during the experimental time course. (PPTX)

S1 File Multiple tables file containing supplementary tables from A to L. **Table A. Primers used for RNA-Seq data validation. Table B. Parameters settings for Galaxy Server bioinformatic tools used for Differential Exon Usage analysis. Table C. Genes differentially expressed in ‘Redkist’ in response to Xap infection**. All the Peach v1.0 genes are reported. **Table D. Genes differentially expressed in ‘JH Hale’ in response to Xap infection**. All the Peach v1.0 genes are reported. **Table E. Genes differentially expressed in ‘Redkist’ and ‘JH Hale’ in response to Xap infection at each time point**. For each gene the v1.0 genome position, matching v2.1 transcript ID, fold change (FC), padj, *Arabidopsis* gene identifiers, gene ontology and function are reported. Down-regulated genes are reported in red. **Table F. Validation of RNA-Seq results by RT-qPCR analysis. Table G. Results of GO-term Enrichment Analysis (Singular Enrichment Analysis)**. Up-regulated and down-regulated genes of both cultivars in response to Xap infection at every time point. **Table H. Significantly enriched GO terms (FDR 0.1) for ‘Redkist’ at 3 hpi with Xap. Table I. Receptor and signal transduction genes differentially expressed in both cultivars at each time point in response to Xap infection. Table J. Transcription factors differentially expressed in both cultivars at each time point in response to Xap infection. Table K. Secondary metabolism genes differentially expressed in both cultivars at each time point in response to Xap infection. Table L. Differential Exon Usage in ‘Redkist’ and ‘JH Hale’ at each time point in response to Xap infection**.
(XLSX)
