# Supplementary material for: Transcriptome reprogramming of resistant and susceptible peach genotypes during Xanthomonas arboricola pv. pruni early leaf infection
Source: PLoS One. 2018 Apr 26;13(4):e0196590. doi: 10.1371/journal.pone.0196590 (PMC5919700; doi:10.1371/journal.pone.0196590)
Supplement: S1 Fig — (PPTX) [file pone.0196590.s001.pptx]

## Slide 1
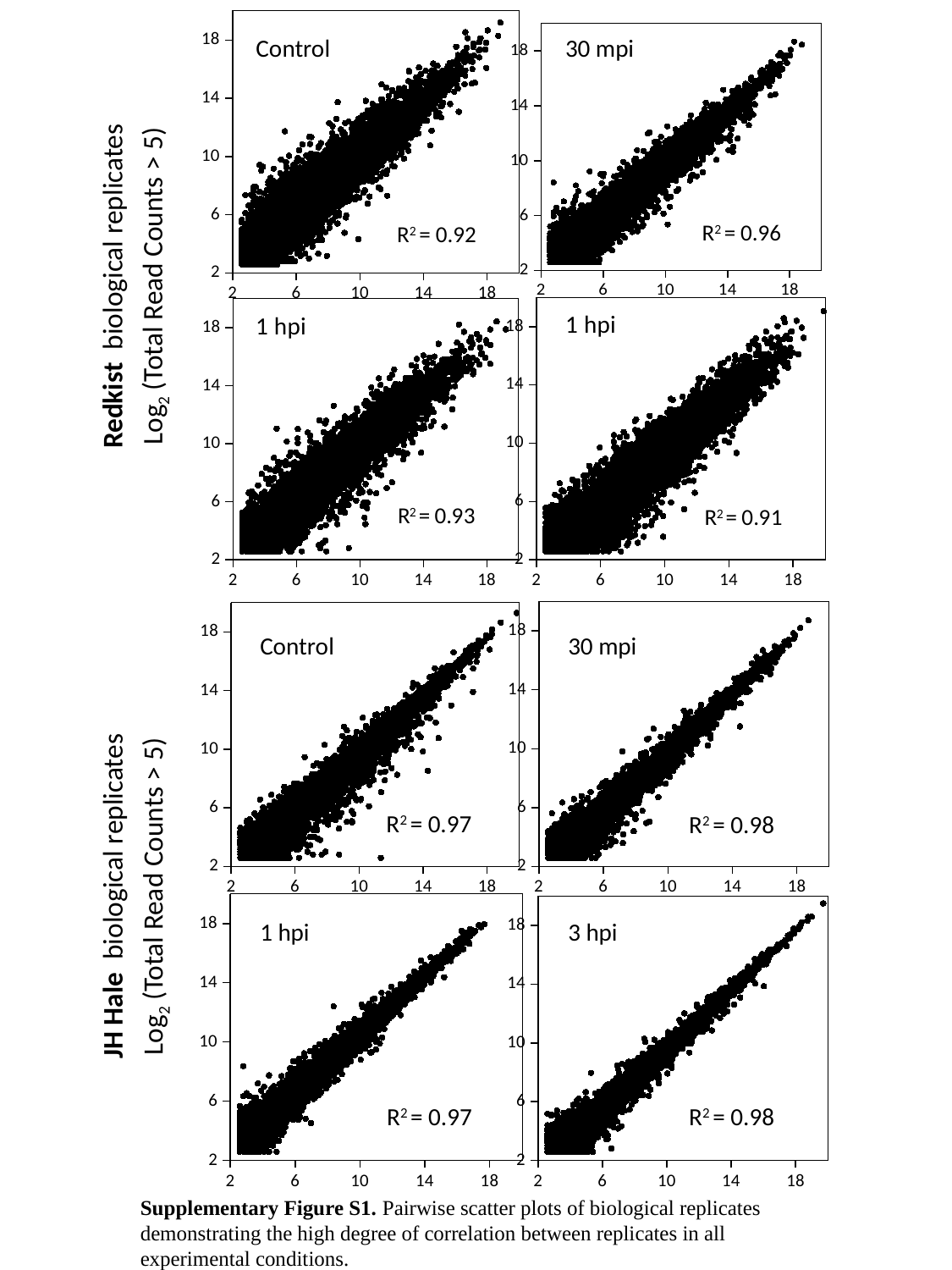

### Chart
| Category | |
|---|---|Control
R2 = 0.92
### Chart
| Category | |
|---|---|30 mpi
R2 = 0.96
### Chart
| Category | |
|---|---|
### Chart
| Category | |
|---|---|Redkist biological replicates
Log2 (Total Read Counts > 5)
### Chart
| Category | |
|---|---|30 mpi
R2 = 0.98
### Chart
| Category | |
|---|---|Control
R2 = 0.97
### Chart
| Category | |
|---|---|1 hpi
R2 = 0.97
### Chart
| Category | |
|---|---|3 hpi
R2 = 0.98
JH Hale biological replicates
Log2 (Total Read Counts > 5)
Supplementary Figure S1. Pairwise scatter plots of biological replicates demonstrating the high degree of correlation between replicates in all experimental conditions.
