# Supplementary figures and images for: Transcriptome reprogramming of resistant and susceptible peach genotypes during Xanthomonas arboricola pv. pruni early leaf infection
Source: PLoS One. 2018 Apr 26;13(4):e0196590. doi: 10.1371/journal.pone.0196590 (PMC5919700; doi:10.1371/journal.pone.0196590)

## Slide 1
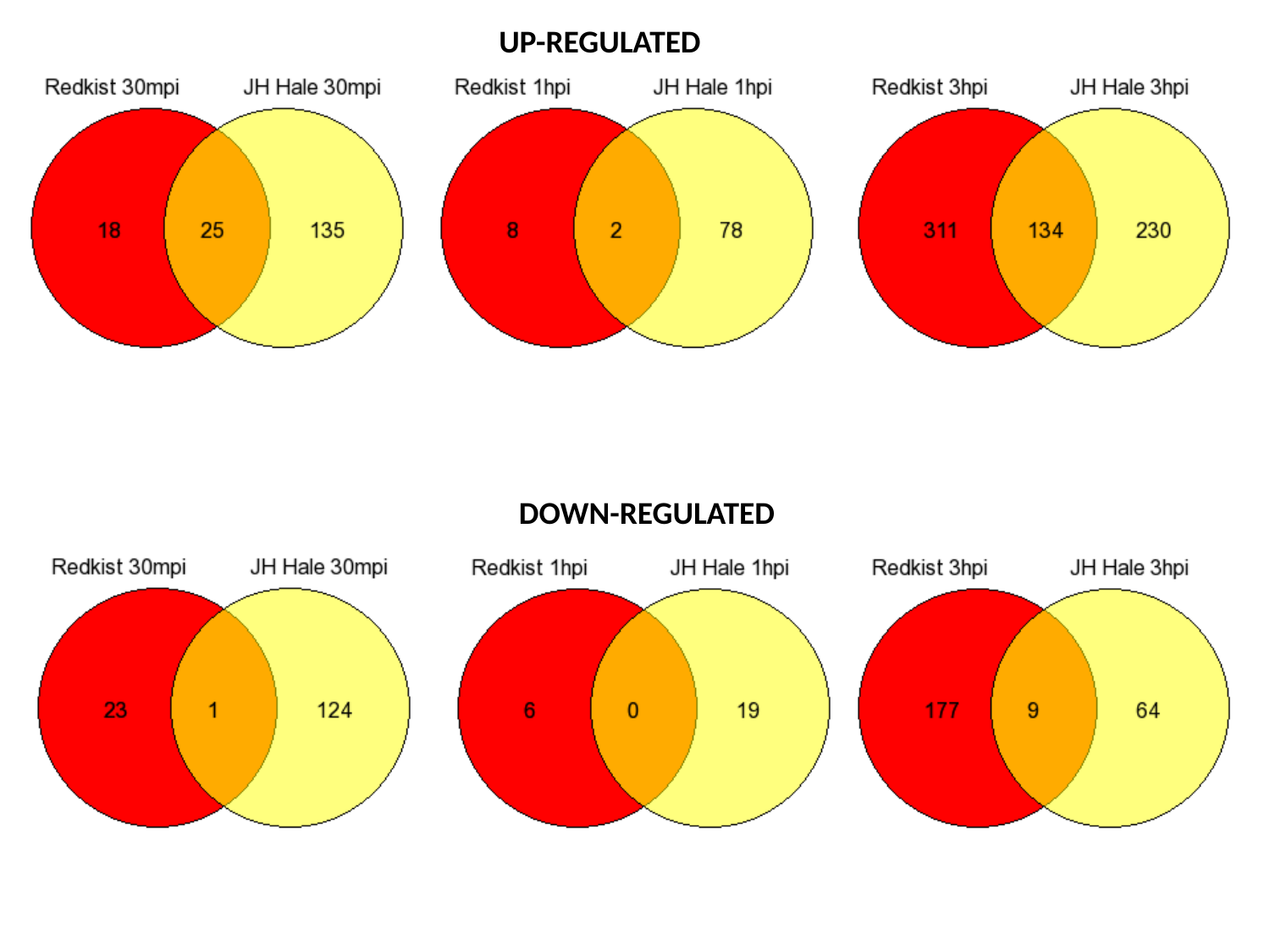

UP-REGULATED
DOWN-REGULATED

Supplement: S2 Fig — (PPTX) [file pone.0196590.s002.pptx]
